# The Impact of Technology-Assisted Interventions on Loneliness in Older Adults: A Meta-Analysis

**DOI:** 10.1093/geroni/igaf122.3081

**Published:** 2025-12-31

**Authors:** Yu-Ping Chang, Ashleigh Holmes, Weijun Wang, Jihnhee Yu

**Affiliations:** University at Buffalo, Buffalo, New York, United States; New York University, New York, New York, United States; University at Buffalo, Buffalo, New York, United States; University at Buffalo, Buffalo, New York, United States

## Abstract

Loneliness and social isolation among older adults are critical public health concerns in the United States, affecting a substantial portion of this population and increasing their risk of cognitive decline, as well as severe mental and physical health consequences. Although recent research highlights the potential of technology-assisted interventions in mitigating these issues, offering promising solutions to enhance social connectivity and well-being, a systematic evaluation of their effectiveness in reducing loneliness among older adults is needed. A meta-analysis of randomized controlled trials was conducted, with a comprehensive literature search of studies published up to November 2024 across four major databases: PubMed, PsycINFO, CINAHL, and Web of Science. Two independent reviewers screened and evaluated the records, resulting in 25 studies meeting the inclusion criteria. These studies examined various types of technology-assisted interventions such as robotic assistants, web-based modules, and mobile applications. Statistical analyses were conducted using R (Version 4.0.2). A heterogeneity analysis using Cochran’s Q test indicated significant variability among studies (Q = 157.21, df = 24, p < 0.001), with an I² estimate of 84.7%, suggesting substantial heterogeneity. Given this, a random-effects meta-analysis was employed to pool effect sizes. The overall effect size (Cohen’s d) was statistically significant at 0.309 (p = 0.007, 95% CI: 0.084–0.534), demonstrating a significant reduction in loneliness among older adults who participated in technology-assisted interventions. These findings underscore the potential of technology as a valuable tool for addressing loneliness. Future research should assess the long-term effectiveness of these interventions and identify best practices for implementation to maximize impact and accessibility.

